# An Exposition of M. Bernard’s Discovery of the Renal Circulation

**Published:** 1850-09

**Authors:** Juriah Harris

**Affiliations:** Georgia, Now in Paris


*An Exposition of M. Bernard's Discovery of the Renal Circulation.* By JURIAH HARRISS, M. D., of Georgia, now in Paris.—The novelty and importance of the discoveries and experiments of M. Bernard have again induced me to report a few of the beautiful results he has obtained, and some of the facts he has established. He has been for several years placing stone after stone upon the great edifice of medicine, of which he is destined to become one of the pilllars of support.

The knowledge of the excessive rapidity with which certain substances, when taken internally, pass out of the economy with the urine, and the ascertained fact that many of them when administered could not be found in the blood, but were identified in the urine, have heretofore been unexplained or have led some into the regions of absurd hypothesis. The mystery was attempted to be explained by supposing that a hidden communication existed between the stomach and kidneys, by which these substances could pass directly from one to the other. This, it is needless to say, is unphysiological and untrue.

M. Bernard has attempted to solve the mystery, and has done so by direct experimentation.

The portal circulation is entirely independent of the pulsations of the heart, and differs from all other venous circulations in the fact that there is an intervening organ between the vein and the heart. The blood, instead of passing directly from the portal vein to the heart, is forced to traverse an organ seated upon its course to the centre of circulation. The portal, and indeed the circulation in all the abdominal viscera, is under the direct influence of the pressure of the abdominal walls. This pressure is essential and indispensable to the circulation of the blood through the abdominal organs, and particularly so to its passage through the portal system. If a small opening be made in the abdomen of an animal, a portion of intestine be withdrawn, and a hydrometer be placed in one of the veins and the intestine returned into the abdomen, an oscilliation will be observed in the tube at each inspiration. The blood is elevated in the tube, because at each inspiration the abdominal cavity is diminished by the descent of the diaphragm and the contractions of the muscles. A notable pressure is thus applied to the contents of the cavity, which is in some degree measured by the elevation in the tube. The pressure is increased or diminished in proportion to the depth of the inspiration. If, after the introduction of the instrument the walls be largely opened, the oscillations will cease, because the pressure is obstructed. In this case the elevation will not only cease, but there will be a counter-current or regurgitation from the inferior vena cava and liver into the vena portæ and mesenteric veins. If prussiate of potash be placed in the inferior cava, by means of a tube, and the abdomen opened, it will regurgitate with the current of blood into the vena porta. This regurgitation occurs in paracentesis abdominis for ascites ; the pressure is removed and hence the syncope. The portal circulation is then entirely dependent upon the abdominal pressure, which last is dependent upon respiration.

During digestion, the quantity of blood in the vena portæ and liver is increased, by the absorption (of considerable albuminous and saccharine principles) which takes place by means of the mesenteric veins. While this act is being accomplished, the blood accumulates in the liver, and distends it, in consequence of the increased supply to the portal system. At this time, either the capilliaries must take on an immense increase of activity to relieve this organ, or the quantity of circulating fluid in the vena portæ must be diminished, before reaching it. There is, in fact, a system of vessels that perform this function. Bernard made this beautiful anatomical discovery in the horse, where it is in reality, very manifest. He has there demonstrated that there is a direct communication of blood-vessels between the vena portæ, and the inferior vena cava, distinct from the communication that exist between the portal and the hepatic veins in the liver. During digestion, when the portal system is engorged with blood, a portion passes through these collateral branches into the vena cava, thereby greatly facilitating the flow from the portal vein. These branches are but small, and ramify upon the vena cava, as do the vasa vasorum upon the intestines, open abruptly into it, by several of them uniting and forming a kind of sinus.

There are in some animals muscular fibres in the walls of the vena cava; they are particularly well marked in the horse. These fibres commence at the diaphragm, and extend down as far as the renal veins; some of them are circular, and others longitudinal. In full digestion, then, the portal system is filled, the liver is increased in volume, and the inferior vena cava and right auricle of the heart, are largely distended by the great increase in the quantity of blood absorbed from the food, by the mesenteric veins. The distension of the vena cava is easily seen in the rabbit. A kind of stagnation occurs in these organs, because the amount of blood is too great to circulate with facility through them. It is at this time that the collateral branches become of great utility, and disgorge the portal system, at the same time that the fibres of the cava, contract and force the blood down to the renal veins, and through them into the kidneys. When these fibres contract, the blood cannot pass up to the heart, because there is already a surplus and stagnation in the right auricle, and must consequently descend. But it may be asked, why does not the blood continue down the iliac and crural veins? It is because, as Bernard has demonstrated, there exists at the opening of the renal veins a valve, which will permit the blood to pass up the vena cava; but when the current is downwards, as in digestion, it closes this vein, and the blood is compelled to pass through the renal veins to the kidneys. At the moment of the downward current, the inferior cava and renal veins can be distinctly seen to pulsate under the influence of the contractions of the muscular fibres. The blood then goes directly to the kidneys for the secretion of urine. Thus, there are many substances absorbed and passed into the portal vein, and from thence to the kidneys, without going around the general circulation.—The obscurity in which the profession has been involved, is thus beautifully dispelled. The rapidity with which certain matters were thrown out of the economy with the urine, and the fact that they could not be identified in the blood, is now easily understood and satisfactorily explained. If prussiate of potash be given to a dog upon an empty stomach, it will be absorbed and carried through the liver, to the heart, and thence to the kidneys. It will be found in the renal arteries, but not in the renal veins. If, on the contrary, it be administered to him during digestion, the stagnation in the vena porta and vena cava, will necessitate the aid of the collateral anastomoses, and the contractions of the vena cava will force it down with the current of blood to the kidneys, where it will be secreted with the ordinary constituents of urine. In this case the prussiate will be found in the renal veins, and but little, if any, in the renal arteries. If an animal drink a large quantity of liquids, a portion will pass directly to the kidneys, without going around the general circulation. This vein, then, resembles the portal in its distribution.

But there is another important question to be answered. How is it, that, when there is a stagnation of blood in the superior part of the inferior vena cava, and a descending current in the lower portion, that the blood can circulate from the inferior extremities? By what route does it reach the heart? Pathology has already, in a measure at least, elucidated this point. It has been observed, and reported, that in cases of obliteration of the superior or inferior vena cava, by a tumor or other cause, that the vena azygos was much distended, and performed the function of the obliterated vein. Though this circulation occurred under a pathological cause, in all cases heretofore reported, the discovery of the renal circulation demonstates that it takes place physiologically. The stagnation of blood in the inferior cava is but a physiological obstruction to the circulation through it to the heart, and as in the case of the pathological cause, the vena azygos uudertakes the work, and conducts the blood to the central organ of circulation. Prior to this time, this was thought to be but an incidental use, arising from an abnormal cause; now, however, it is demonstrated to be a natural and physiological function, that this vein daily performs. The office of this vein is then to carry on the circulation between the inferior extremities and the heart, during digestion.

During full digestion, there is no circulation in the kidneys. The blood is carried on by both veins and arteries, and consequently has no means of exit. It is at this moment that the urine changes its character according to the nature of the food taken. If the food be azotized, the urine will be acid, and if not azotized, it will be alkaline. In an animal fasting (a rabbit) the blood reaches the kidneys alone by the arteries, and the urine will be clear and acid. But if ordinary food (non-azotized) be given him, the blood will reach the kidneys, both by the veins and arteries, and the urine will be turbid and alkaline. The phosphate of soda, that exists in abundance in the urine of a rabbit, after digestion, does not exist in the blood, but is formed in the pelvis of the kidneys, in consequence of the combination of the two kinds of urine that meet at this point. The acids of the one, unite with the alkaline principles of the other.

If medicine be administered during digestion, a great portion of it at least will pass down the renal veins to the kidneys, and be thrown off with the urine ; and thus it is that a dose of poison, which, during abstinence, would probably prove fatal, may be thrown out of the economy, during digestion, without producing serious accidents. Belladonna, or its active principle, atrophine, will not readily kill an animal during digestion, but will do so speedily when fasting. Hence we can conceive the reason why a rabbit is not killed by these strong narcotics, when the same dose will be fatal to a dog. It is because a rabbit is really always digesting ; his stomach may almost be said never to be empty, even when made to fast for several days. If this animal be kept from eating a long time, he will be killed as readily as the dog.

A dose of poison, to kill an animal, must be much larger during digestion, than during abstinence.

In reptiles, there is a peculiar arrangement of the vena portæ. There exists in them two venæ portæ : the one that is found in other animals and distributed to the liver, and the other that passes to the kidneys, and may be styled the renal vena portæ. This arrangement in man is assimilated by the renal circulation.

